# Dispersion Engineering of Silicon Nitride Microresonators via Reconstructable SU-8 Polymer Cladding

**DOI:** 10.3390/mi13030454

**Published:** 2022-03-17

**Authors:** Shang-Pu Wang, Tien-Hsiang Lee, You-Yuan Chen, Pei-Hsun Wang

**Affiliations:** Department of Optics and Photonics, National Central University, Taoyuan City 32001, Taiwan; wsp870521@gmail.com (S.-P.W.); thmos10933@gmail.com (T.-H.L.); s108226072@dop.ncu.edu.tw (Y.-Y.C.)

**Keywords:** group velocity dispersion, optical waveguide, dispersion engineering, reconstructable cladding layer, microresonator

## Abstract

In this work, we propose a novel way to flexibly engineer the waveguide dispersion by patterning the cladding of waveguide microresonators. Experimentally, we demonstrate silicon nitride waveguides with air-, oxide-, and SU-8 polymer-cladding layers and compare the corresponding waveguide dispersion. By integrating SU-8 polymer as the outer cladding layer, the waveguide dispersion can be tuned from −143 to −257 ps/nm/km. Through the simple, conventional polymer stripping process, we reconstruct the waveguide dispersion back to that of the original air-cladded device without significantly impacting the quality factor of resonators. This work provides the potential to design the waveguide dispersion in normal and anomalous regimes within an integrated photonic circuit.

## 1. Introduction

Group-velocity dispersion (GVD) of optical waveguides play an important role in many optical applications of photonics, such as nonlinear optics [1], ultrafast optic [2], and high-speed optical communication [3]. Efficient and precise control of GVD becomes increasingly crucial for most applications. For instance, in optical communication, low dispersion is preferred to maintain the signal quality within a wide bandwidth, and dispersion compensation strategies are needed to cancel the inherent material dispersion [4]. In nonlinear optics (e.g., parametric generation), anomalous dispersion is required to initiate modulational instability [5]. Traditionally, dispersion is engineered by designing the waveguide geometries (e.g., height and width) [6,7]. Normal to anomalous dispersion can be achieved by tailoring the height of a silicon nitride (Si_3_N_4_) waveguide from 500 nm to 1000 nm [6]. Although this geometrical design has been widely applied to compensate for the material dispersion and to achieve desirable dispersion, this method requires dedicated fabrication control. Moreover, it loses flexibility in the design of integrated waveguides. In the meantime, several alternative methods have been proposed to control the waveguide dispersion. By depositing hafnium oxide thin film on top of the Si_3_N_4_ waveguide via atomic layer deposition (ALD), GVD can be tuned from 49.2 ps/nm/km to 70.3 ps/nm/km [8]. However, this needs an additional deposition process, and the dispersion is globally defined for the entire chip, lacking the flexibility in the design of multi-project wafers (MPW). Another way to tailor the waveguide dispersion is to harness the spatial-mode-coupling by introducing coupling through the auxiliary resonator [9]. This releases the waveguide geometry restriction and provides a more flexible way to engineer the waveguide dispersion. Nevertheless, the coupled structures complicate the waveguide operation, e.g., frequency comb generation in microresonators [10], and only local dispersion can be altered at a specific wavelength. Moreover, the coupling condition is sensitive to operating conditions like optical power and cavity temperature. More recently, locally patterning geometries have been introduced to pattern the dispersion of a specific device in an integrated chip. Through locally removing the oxide cladding on microresonators, the waveguide dispersion of lithium niobate (LN) resonators can be individually designed at both normal and anomalous within the same chip [11]. However, it may induce surface roughness of waveguides when the cladding oxide layer is removed through a photolithography step followed by hydrofluoric acid (HF) wet etching. This additional process increases the waveguide loss and reduces resonators’ available quality (Q) factor. A few methods locally tailor the dispersion by post-trimmings [12] or integrating cladding-modulated Bragg gratings along the waveguides [13]. Unfortunately, these methods either require a well-controlled dry-etching process or need complicated grating structures in the layout design. More importantly, all of the demonstrated processes are not reconstructable–the dispersion is not changeable once the fabrication process determines the waveguide geometries and patterning.

In this work, we propose a simple, flexible, and reconstructable way to tailor the dispersion by cladding polymer on the Si_3_N_4_ waveguide. We take SU-8, a commonly used negative photoresist, as the polymer-cladding. This material has been widely adapted in 3D microstructures with low-cost and easy-processing fabrication [14,15,16,17]. Furthermore, due to the low optical absorption at 1550 nm, the SU-8 photoresist has also been integrated as a polymeric waveguide [18,19]. The polymer-cladding can be easily patterned and removed without damaging the waveguide surface, which helps program the dispersion of different waveguides/resonators individually within the same chip for multi-purposes. Our work results in several new findings. First, we show the feasibility of tuning the dispersion by a patternable polymer cladding. Experimentally, the dispersion can be tailored between −143 ps/nm/km and −257 ps/nm/km of a 3 µm* 500 nm (cross-section) Si_3_N_4_ waveguide. Second, the waveguide dispersion of air-, oxide-, and SU-8 polymer-cladding layers are compared, qualitatively agreeing with the simulated data. Last, we show that the dispersion can be reconstructed by removing the cladding layer without impacting the quality factor. The dispersion engineering demonstrated here provides the versatility to tune waveguide dispersion for linear and nonlinear integrated photonics applications.

## 2. Dispersion Simulation

**Figure 1:**
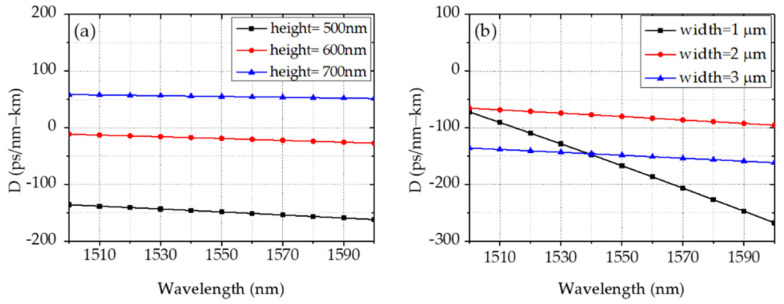
Waveguide dispersion with different geometries. (**a**) Dispersion of waveguides with varying heights. (**b**) Dispersion of waveguides with varying widths.

We first geometrically simulate the waveguide dispersion of the fundamental transverse electric (TE) mode for a silicon nitride waveguide based on the finite element method (FEM) at 1550 nm [20]. The numerical results are shown in Figure 1a by varying heights from 500 nm to 700 nm with a fixed width of 3 μm and in Figure 1b by varying widths from 1 μm to 3 μm with a fixed height of 500 nm. We can see that GVD moves from normal to anomalous by increasing the waveguide height above 700 nm, in agreement with the previous literature [6]. A smoother curve with less wavelength-dependency can be obtained by increasing the waveguide width from 1 μm to 3 μm.

**Figure 2:**
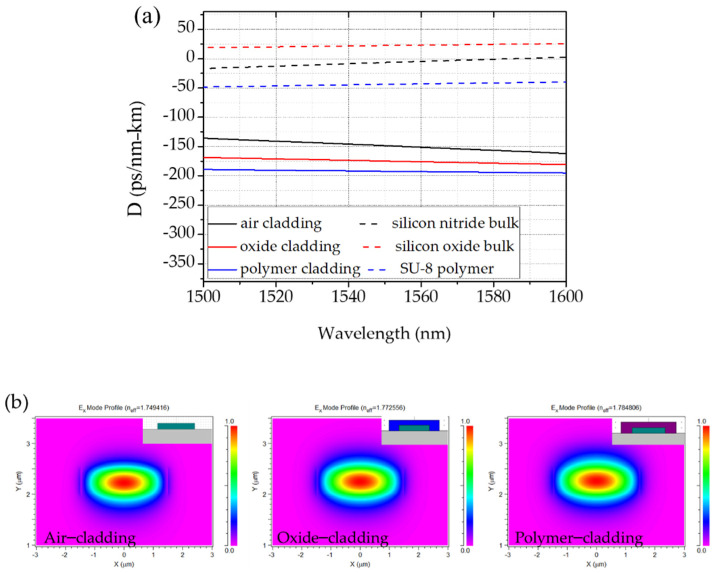
(**a**) Waveguide dispersion of air-, oxide-, and SU-8 polymer-cladding with 500 nm height and 3 μm width (solid lines) and material dispersion of silicon nitride, silicon oxide, and SU-8 polymer (dashed lines). (**b**) Field distribution of the waveguide without cladding layer, with oxide-cladding layer, and with polymer-cladding layer.

To address the cladding effect on the waveguide dispersion, we show the simulated dispersion with air-, oxide-, and SU-8 polymer-cladding in Figure 2. With a 3 μm * 500 nm (cross-section) Si_3_N_4_ waveguide, air-cladding exhibits weaker normal dispersion than the oxide-cladded and SU-8 polymer-cladded waveguide. We should note that the polymer cladding can be easily patterned by spinning and removing SU-8 through the conventional lithography process. Thus, the dispersion can be adjusted by selecting the proper coverage ratio between polymer and air cladding. It offers a flexible way to achieve targeted dispersion. In addition, this polymer cladding shows deeper in normal dispersion regime than that offered by an oxide-cladded waveguide, providing more tunability for applications requiring normal dispersion [21].

**Figure 3:**
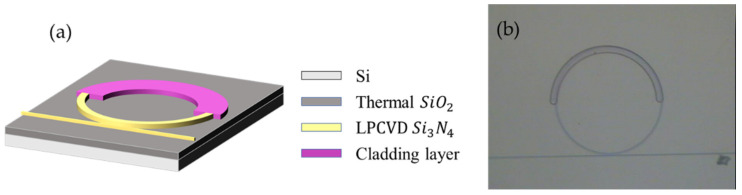
(**a**) The exemplary schematics and (**b**) image of the fabricated microresonator of polymer-cladding on the microresonator with 50% coverage.

To further illustrate this concept, Figure 3 shows the exemplary schematics and the microscope image of the fabricated resonator with 50% coverage of polymer cladding. To avoid unwanted scattering loss and coupling change, the polymer-cladding can be chosen not to cover the coupling regime between the resonators and bus waveguide; or an air void may be found between the narrow gap. This results in excess coupling loss and degrading the loaded quality factor of the resonator [22].

**Figure 4:**
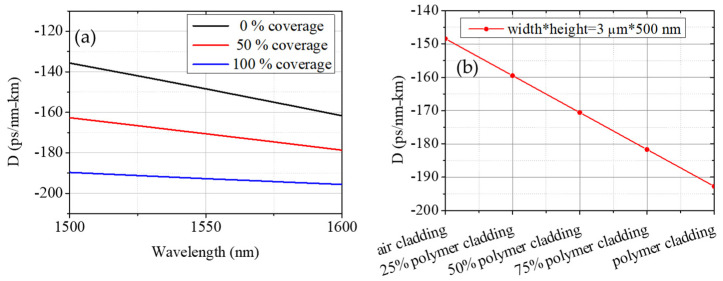
(**a**) Dispersion with 0 %, 50%, and 100 % coverage of polymer-cladding on the microresonator with 500 nm height and 3 μm width. (**b**) The corresponding dispersion dependency on the cladding coverage at 1550 nm.

The simulated GVD with 0 %, 50%, and 100 % coverage of the polymer-cladding on the microresonator is shown in Figure 4a. The cross-section of the waveguide is set at 500 nm height and 3 μm width. Based on the selected coverage of the polymer-cladding, the dispersion can be interpolated between that of air- and polymer-cladding. This relieves the previous geometrical requirement on the specific waveguide dimension.

Figure 4b shows the corresponding dispersion around 1550 nm by changing the coverage of the SU-8 polymer-cladding. A 3 μm * 500 nm waveguide dispersion can be tailored from −148 to −192 ps/km/nm by covering the polymer-cladding from 0% to 100%. We can see that, for both waveguide geometries, the dispersion is engineered toward to weaker normal regime by reducing the polymer-cladding coverage. This again exhibits the versatility to design waveguide dispersion in an integrated device.

## 3. Device Fabrication and Experimental Setup

**Figure 5:**
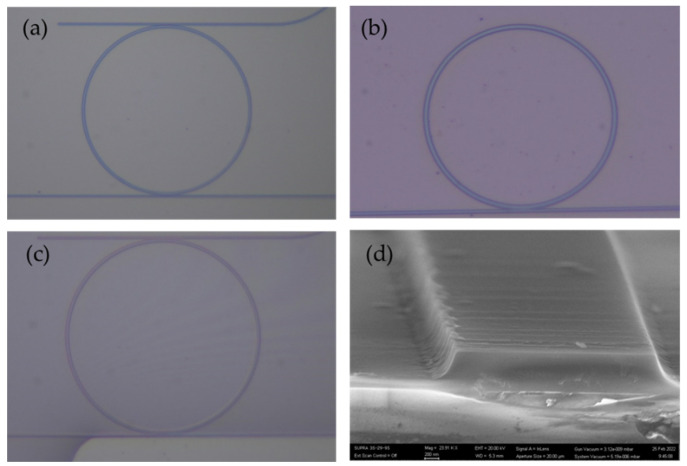
Images of the fabricated microresonators (**a**) without cladding layer, (**b**) with oxide cladding layer, and (**c**) with SU-8 polymer-cladding layer. (**d**) The SEM image of the air-cladded waveguide.

In fabrication, a 4 μm thick silicon oxide (SiO_2_) layer was thermally grown on silicon wafers in a diffusion furnace (Semiconductor Wafer, Inc., Hsinchu, Taiwan). Next, a 500 nm Si_3_N_4_ film was deposited with low-pressure chemical vapor deposition (LPCVD). After Si_3_N_4_ deposition, e-beam lithography (EBL, ELIONIX, Tokyo, Japan) is used to pattern the waveguide resonators with ma-N 2405, a negative-tone resist. The devices were then dry-etched in a high-density plasma etching tool (Unaxis/Nextral 860L, Grenoble, France). For the oxide-cladded device, a 1 μm SiO_2_ layer was deposited upon the Si_3_N_4_ resonators with plasma-enhanced chemical vapor deposition (SAMCO PD-220N, SAMCO, Kyoto, Japan) and annealed at 1050 °C for 10 h to improve the quality factor of the resonators. As for the polymer-cladded device, SU-8 GM1040 (Gersteltec, Pully, Switzerland) was spin-coated on the air-cladded microresonator with 135 °C baking for two hours. The fabricated devices are shown in Figure 5.

**Figure 6:**
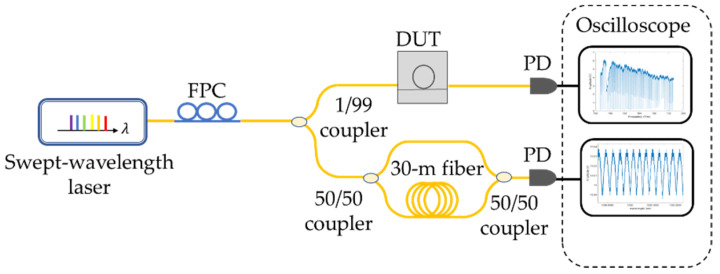
Scheme of the experimental setup.

Figure 6 shows the experimental setup for characterizing waveguide dispersion. A C-band, tunable laser (SANTEC TSL-550, Santec Corporation, Aichi, Japan) is used for transmission measurement. The power in the waveguide is kept below 0 dBm to avoid unwanted thermo-optical effect for the fabricated resonators. At the DUT side, a pair of lensed fibers were used to couple light into and out of the integrated Si_3_N_4_ waveguides. We measured the waveguide-TE mode in which the electric field was polarized parallel to the waveguide. Then, 1% power from the laser was split to a fiber-based interferometry system with a 30 m optical path difference. The generated interference fringes showed a periodicity around 6.89 MHz, exceeding the available frequency resolution from the tunable laser system. These reference markers not only calibrate the frequency instability from the swept-wavelength laser [23] but also extract Q factors, especially from ultra-high-Q devices [24].

## 4. Dispersion Measurements

For waveguide microresonators, the dispersion leads to the frequency difference between the adjacent FSRs. This value is typically small for a waveguide with weak dispersion. The dispersion parameter *D* with resonance frequencies can be expressed as [25,26]:

(1) $$D=\frac{2\pi c}{4\pi^{2}\lambda^{2}R\cdot FSR^{3}}\frac{dFSR}{dm}\;$$

where *FSR* is the free-spectral-range of microresonators, *c* is the speed of light, *R* is the radius of the microresonator, *m* is the azimuthal mode number, and λ is the optical wavelength. $\frac{dFSR}{dm}$ describes the frequency difference in FSR.

**Figure 7:**
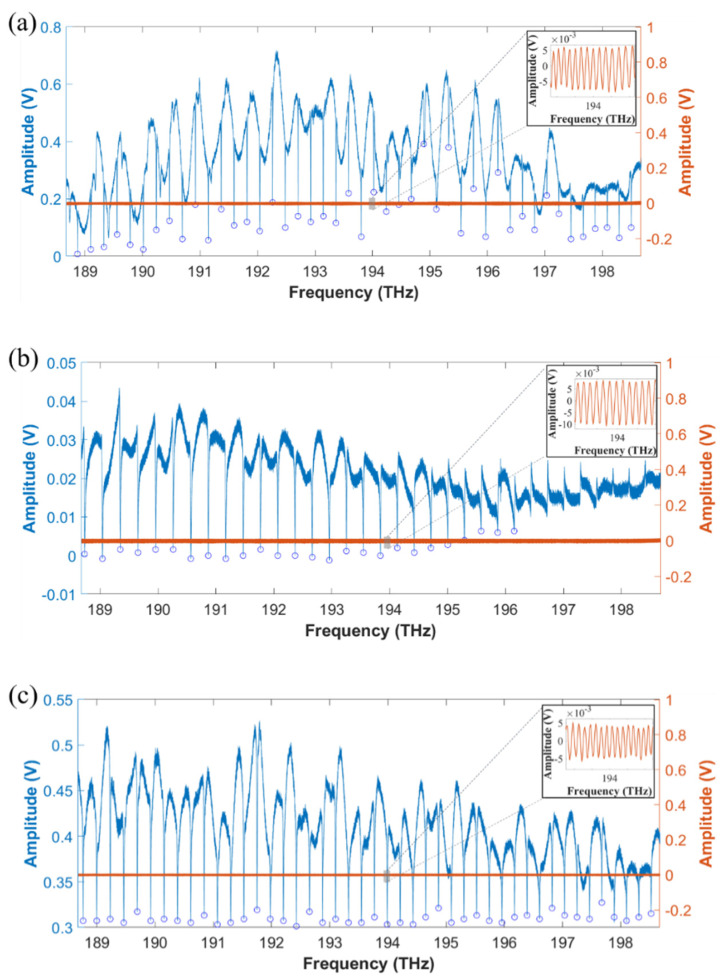
Transmission spectra of the microresonators (blue line) (**a**) without cladding, (**b**) with oxide-cladding, and (**c**) with polymer-cladding. The corresponding interference fringes from the interferometer system are simultaneously recorded for frequency calibration (orange line). The zoom-in spectrum of the interference fringes is shown in the insets.

The measured results of air-cladded, oxide-cladded, and polymer-cladded waveguide resonators are shown in Figure 7a–c. The fitted curve at around 1550 nm exhibits Lorentzian-shape with the extracted intrinsic quality factor $Q_{i}\sim 1.6\times 10^{5}$ (air-cladded), $Q_{i}\sim 2.4\times 10^{4}$ (oxide-cladded), and $Q_{i}\sim 1.1\times 10^{4}$ (polymer-cladded). The insets show the corresponding zoom-in interference fringes from the interferometer system.

**Figure 8:**
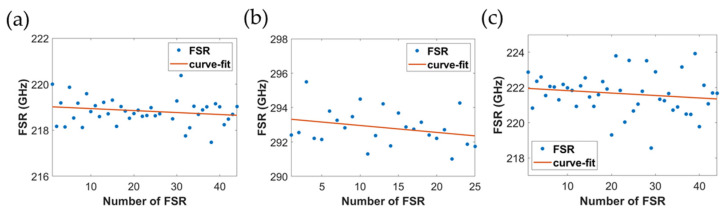
Distribution of FSRs of the fundamental TE mode for (**a**) air-cladded, (**b**) oxide-cladded, and (**c**) polymer-cladded waveguide microresonators.

As we shown in Figure 8a–c. For the air-cladded device, the radius of the microresonator is 100 μm, and the fitted FSR difference is 7.31 MHz/FSR, while for the oxide-cladded device, the radius of the microresonator is 75 μm with the measured FSR difference of 15.7 MHz/FSR. As for the polymer-cladded device, 1 μm SU-8 polymer layer was coated on the originally air-cladded device, showing the fitted FSR difference of 14.7 MHz/FSR. The slight difference in the averaged FSRs was due to the difference in group index n_g_ between air-cladded (n_g_ ≈ 2.0716) and polymer-cladded devices (n_g_ ≈ 2.0378).

**Figure 9:**
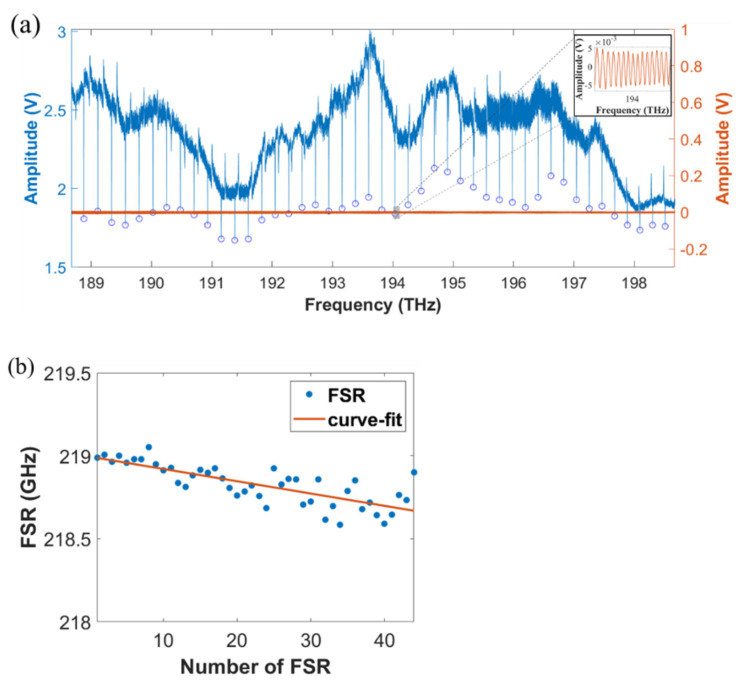
(**a**) Transmission spectrum of the microresonator (blue line) with cladded layer stripping. The corresponding interference fringes from the interferometer system (orange line). The zoom-in spectrum of the interference fringes is shown in the inset. (**b**) Distribution of FSRs.

To show the reconstructability of the polymer cladding layer, we then stripped the polymer-cladding layer by acetone (100 °C 10 min). The measured result is shown in Figure 9a. The extracted intrinsic quality factor is around $Q_{i}\sim 1.3\times 10^{5}$, similar to that measured with the original air-cladded device. The fitted difference of FSR was 7.40 MHz/FSR.

**Figure 10:**
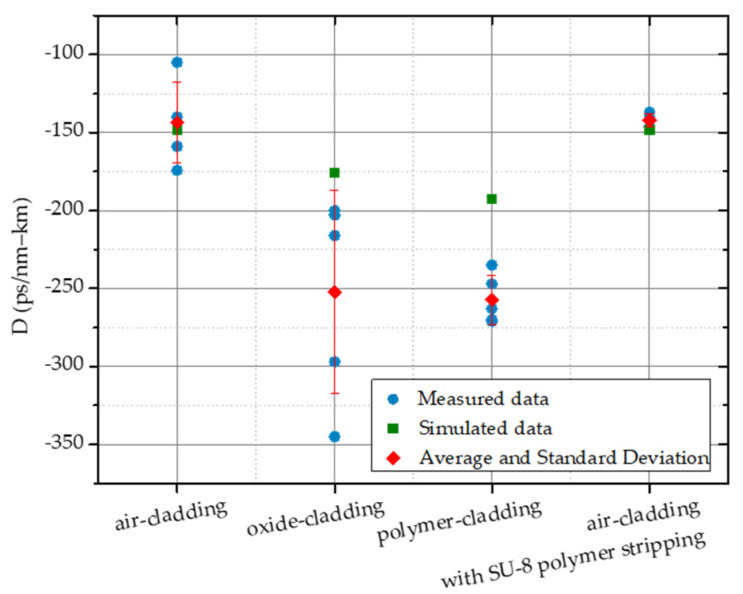
Simulated and measured dispersion parameters for air-, oxide-, SU-8 polymer-cladding, and air-cladded resonators with polymer stripping.

We show the comparison of the measured dispersion of air-, oxide-, SU-8 polymer-cladded, and air-cladded resonators with polymer stripping in Figure 10. The measured data were in qualitative agreement with the simulated data. For the air-cladded device, the measured dispersion parameter was −143 ± 26 ps/nm-km with the simulated value −148 ps/nm-km. In contrast, the measured dispersion parameter for the SU-8 polymer-cladded device was −257 ± 16 ps/nm-km with the simulated value −192 ps/nm-km. Higher-order terms are ignored here. The polymer-cladding effectively pushes the dispersion deeply into the normal regime, as observed in the simulation. As mentioned, the dispersion can be further engineered by designing a proper coverage layout to target the desired dispersion between air- and polymer-cladding. For the oxide-cladded device, normal dispersion −252 ± 65 ps/nm-km can be achieved with the simulated value −176 ps/nm-km. The larger variation may have come from the relatively smaller radius and, therefore larger FSR. For the device with polymer stripping, the measured dispersion parameter is −142 ± 4 ps/nm-km, which is close to that of the originally air-cladded device. 

**Figure 11:**
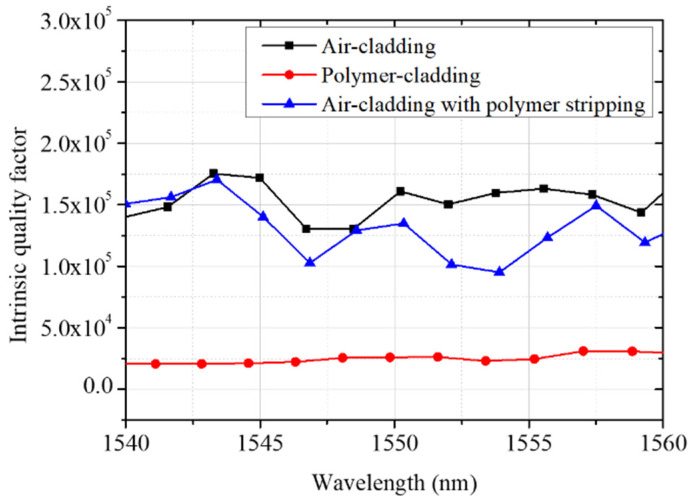
The distribution of the intrinsic quality factor $Q_{i}$ for air-cladded, SU-8 polymer-cladded, and air-cladded resonators with polymer stripping.

Figure 11 shows the distribution of the intrinsic quality factor $Q_{i}$ for air-cladded, SU-8 polymer-cladded, and air-cladded resonators with polymer stripping. With SU-8 polymer cladding, $Q_{i}$ decreases by order of magnitude due to the optical loss in the cladding layer. However, after polymer stripping, the intrinsic quality factor can be retrieved, comparable to that of the original air-cladded device.

## 5. Discussion

This section further discusses dispersion engineering with SU-8 polymer cladding. First, we should note here that, although this work shows the tunability in the normal dispersion regime only, it can also be applied in the anomalous regime with a thicker Si_3_N_4_ waveguide (>700 nm), offering a way to elegantly engineer the dispersion from anomalous to normal for applications requiring low/zero dispersion. Furthermore, the dispersion can be constructed by removing the cladded polymer and depositing again with different coverages.

Second, in addition to the widely used SU-8 polymer, polymethylmethacrylate (PMMA) could be an alternative cladding material for patterning dispersion. However, PMMA can only be patterned by etching, femtosecond-laser writing [27], or with nanoimprint lithography (NIL) [28]. All of these may degrade the optical properties. SU-8 is suitable since the low optical absorption at 1550 nm and can be easily patterned by conventional lithography, which is fast, simple, and cost-effective.

Last, this novel scheme can be applied to any integrated waveguides. For instance, dispersion engineering of a waveguide-based Mach-Zehnder interferometer provides a better figure of merit for index sensing [29]. Moreover, it could help achieve phase-matching conditions for a second-harmonic generation [30] or four-wave-mixing in a straight waveguide [31].

## 6. Conclusions

In summary, we propose a novel way to engineer the waveguide dispersion by patterning the outer cladding with SU-8 polymer. With the aid of the polymer-cladding layer, the dispersion of a Si_3_N_4_ waveguide can be tuned from −143 to −257 ps/nm-km. In addition, we show the capability to reconfigure the waveguide dispersion with the conventional polymer stripping process. This work shows the potential to tailor dispersion at a specific value with different cladding coverages and the flexibility to reconfigure waveguide dispersion individually for multi-purpose applications in an integrated device.

## Data Availability

Not applicable.

## References

[1] Turner A.C., Manolatou C., Schmidt B.S., Lipson M., Foster M.A., Sharping J.E., Gaeta A.L. (2006). Tailored anomalous group-velocity dispersion in silicon channel waveguides. Opt. Express.

[2] Tan D.T., Agarwal A.M., Kimerling L.C. (2015). Nonlinear photonic waveguides for on-chip optical pulse compression. Laser Photonics Rev..

[3] Eldada L. (2004). Optical communication components. Rev. Sci. Instrum..

[4] Guo Y., Jafari Z., Xu L., Bao C., Liao P., Li G., Agarwal A.M., Kimerling L.C., Michel J., Willner A.E. (2019). Ultra-flat dispersion in an integrated waveguide with five and six zero-dispersion wavelengths for mid-infrared photonics. Photonics Res..

[5] Tai K., Hasegawa A., Tomita A. (1986). Observation of modulational instability in optical fibers. Phys. Rev. Lett..

[6] Tan D.T.H., Ikeda K., Sun P.C., Fainman Y. (2010). Group velocity dispersion and self phase modulation in silicon nitride waveguides. Appl. Phys. Lett..

[7] Boggio J.C., Bodenmüller D., Fremberg T., Haynes R., Roth M., Eisermann R., Lisker M., Zimmermann L., Böhm M. (2014). Dispersion engineered silicon nitride waveguides by geometrical and refractive-index optimization. JOSA B.

[8] Riemensberger J., Hartinger K., Herr T., Brasch V., Holzwarth R., Kippenberg T.J. (2012). Dispersion engineering of thick high-Q silicon nitride ring-resonators via atomic layer deposition. Opt. Express.

[9] Li Y., Li J., Huo Y., Chen M., Yang S., Chen H. (2018). Spatial-mode-coupling-based dispersion engineering for integrated optical waveguide. Opt. Express.

[10] Kim S., Han K., Wang C., Jaramillo-Villegas J.A., Xue X., Bao C., Xuan Y., Leaird D.E., Weiner A.M., Qi M. (2017). Dispersion engineering and frequency comb generation in thin silicon nitride concentric microresonators. Nat. Commun..

[11] Wang C., Zhang M., Yu M., Zhu R., Hu H., Loncar M. (2019). Monolithic lithium niobate photonic circuits for Kerr frequency comb generation and modulation. Nat. Commun..

[12] Moille G., Westly D., Orji N.G., Srinivasan K. (2021). Tailoring broadband Kerr soliton microcombs via post-fabrication tuning of the geometric dispersion. Appl. Phys. Lett..

[13] Sahin E., Ooi K., Png C., Tan D. (2017). Large, scalable dispersion engineering using cladding-modulated Bragg gratings on a silicon chip. Appl. Phys. Lett..

[14] Mata A., Fleischman A.J., Roy S. (2006). Fabrication of multi-layer SU-8 microstructures. J. Micromech. Microeng..

[15] Abgrall P., Conedera V., Camon H., Gue A.M., Nguyen N.T. (2007). SU-8 as a structural material for labs-on-chips and microelectromechanical systems. Electrophoresis.

[16] Shang X., Ke M., Wang Y., Lancaster M.J. (2012). WR-3 Band Waveguides and Filters Fabricated Using SU8 Photoresist Micromachining Technology. IEEE Trans. Terahertz Sci. Technol..

[17] Pinto V.C., Sousa P.J., Cardoso V.F., Minas G. (2014). Optimized SU-8 processing for low-cost microstructures fabrication without cleanroom facilities. Micromachines.

[18] Ariannejad M.M., Amiri I.S., Ahmad H., Yupapin P. (2018). A large free spectral range of 74.92 GHz in comb peaks generated by SU-8 polymer micro-ring resonators: Simulation and experiment. Laser Phys..

[19] Dai D., Yang B., Yang L., Sheng Z., He S. (2009). Compact microracetrack resonator devices based on small SU-8 polymer strip waveguides. IEEE Photonics Technol. Lett..

[20] (2007). RSoft FemSIM.

[21] Xue X., Wang P.H., Xuan Y., Qi M., Weiner A.M. (2017). Microresonator Kerr frequency combs with high conversion efficiency. Laser Photonics Rev..

[22] Schwelb O. (2004). Transmission, group delay, and dispersion in single-ring optical resonators and add/drop filters-a tutorial overview. J. Lightwave Technol..

[23] Twayana K., Ye Z., Helgason O.B., Vijayan K., Karlsson M., Torres-Company V. (2021). Frequency-comb-calibrated swept-wavelength interferometry. Opt. Express.

[24] Jin W., Yang Q.-F., Chang L., Shen B., Wang H., Leal M.A., Wu L., Gao M., Feshali A., Paniccia M. (2021). Hertz-linewidth semiconductor lasers using CMOS-ready ultra-high-Q microresonators. Nat. Photonics.

[25] Fujii S., Tanabe T. (2020). Dispersion engineering and measurement of whispering gallery mode microresonator for Kerr frequency comb generation. Nanophotonics.

[26] Hong Y., Hong Y., Hong J., Lu G.-W. (2021). Dispersion Optimization of Silicon Nitride Waveguides for Efficient Four-Wave Mixing. Photonics.

[27] Homoelle D., Wielandy S., Gaeta A.L., Borrelli N., Smith C. (1999). Infrared photosensitivity in silica glasses exposed to femtosecond laser pulses. Opt. Lett..

[28] Nordström M., Zauner D.A., Boisen A., Hübner J. (2007). Single-mode waveguides with SU-8 polymer core and cladding for MOEMS applications. J. Lightwave Technol..

[29] Lee D.E., Lee Y.J., Shin E., Kwon S.-H. (2017). Mach-Zehnder interferometer refractive index sensor based on a plasmonic channel waveguide. Sensors.

[30] Nitiss E., Zabelich B., Yakar O., Liu J., Wang R.N., Kippenberg T.J., Brès C.-S. (2020). Broadband quasi-phase-matching in dispersion-engineered all-optically poled silicon nitride waveguides. Photonics Res..

[31] Driscoll J.B., Ophir N., Grote R.R., Dadap J.I., Panoiu N.C., Bergman K., Osgood R.M. (2012). Width-modulation of Si photonic wires for quasi-phase-matching of four-wave-mixing: Experimental and theoretical demonstration. Opt. Express.

